# Vancomycin levels are frequently subtherapeutic during continuous venovenous hemodialysis (CVVHD) 

**DOI:** 10.5414/CN106993

**Published:** 2011-07-13

**Authors:** F. Perry Wilson, Jeffrey S. Berns

**Affiliations:** University of Pennsylvania Health System, Department of Medicine, Renal, Electrolyte and Hypertension Division, Philadelphia, PA, USA

**Keywords:** continuous renal replacement therapy, pharmacokinetics, vancomycin, median inhibitory concentration, clearance

## Abstract

Abstract. Continuous renal replacement therapy (CRRT) is frequently used in the intensive care setting for the treatment of acute kidney injury. Dosing guidelines for many commonly used antibiotics were established during intermittent dialysis or in studies examining CRRT at lower blood and dialysis flow rates than are used in common practice. Herein we present data demonstrating frequent subtherapeutic levels of vancomycin in a population of patients on CRRT. Nephrology trainees should be educated as to the risks of under-dosing antibiotics in this population.

## Neph-Ed material 

Continuous renal replacement therapy (CRRT) is commonly utilized in the treatment of acute kidney injury in critically ill patients [1]. Medication dosing recommendations for patients on CRRT are often either empiric, abstracted from studies of intermittent dialysis, based on pharmacokinetic studies of continuous dialysis using lower blood and dialysate flow rates than are possible and often used with newer CRRT machines[2, 3], or extrapolated from one CRRT modality to another. Subtherapeutic antibiosis may lead to adverse clinical outcomes, and nephrology trainees need to be aware for the potential of under-dosing this population. We conducted a retrospective cohort study to examine the serum concentrations of vancomycin in patients treated with continuous venovenous hemodialysis (CVVHD) and to examine vancomycin dosing and monitoring practices in our institution. 

We identified all patients receiving CVVHD at our institution (a large, metropolitan teaching hospital) from 7/1/2007 to 12/31/2007 (N = 70). Patients were analyzed in aggregate and in subgroups by medical or surgical intensive care unit. For analysis of vancomycin dosing and monitoring practices, we studied only patients who had received 2 or more vancomycin doses during a continuous period of CVVHD treatment (n = 40). Patients were prescribed vancomycin in doses that were typically 1.0 – 1.5 g initially with the intent of using subsequent random levels to guide further dosing – a “dose by level” strategy. Our standard CVVHD protocol includes Qb = 300 ml/min and QD = 2,000 – 3,000 ml/h. 

Demographic and pharmacologic data are presented in Table 1. 

In-hospital mortality was 64% among medical intensive care unit (MICU) patients (n = 9) and 67% among surgical (SICU) patients (n = 31) (p = 0.91). The median hospital length of stay was 22 days (IQ range 6.5 – 46 days) for MICU patients and 27 days (IQ range 20 – 47 days) for SICU patients (p = 0.24). 

Mean vancomycin dosages were similar in both patient groups; 10.7 ± 5.81 mg/kg/d in MICU patients and 14.0 ± 8.03 mg/kg/d in SICU patients (p = 0.16). 

The therapeutic vancomycin level was defined as a level ≥ 15µg/dl, consistent with studies suggesting that higher vancomycin levels may be necessary for treatment of methicillin-resistant Staph. aureus (MRSA)[4, 5, 6]. As defined, 44% of levels obtained in MICU patients and 49% of levels obtained in SICU patients were subtherapeutic. The median time to re-dosing after a subtherapeutic level was 6 h (IQ range 3.5 – 12.0 h) in MICU patients and 4 h (IQ range 2.9 – 12.8 h) in SICU patients. 22% of MICU patients and 29% of SICU patients did not have a vancomycin level checked during CVVHD therapy despite receiving treatment for at least 2 doses and at least 3 days. 

We created a scatter plot that relates serum vancomycin concentration to the time from the most recent dose. This gives a sense of the rapidity with which vancomycin levels can become subtherapeutic, often falling to such a level within 24 h of dosing. 

The chief strength of the study lies in the relatively large population in whom serum drug levels were available. In addition, our institution utilizes an electronic ordering system for drugs and dialysis, making selection bias less likely. The study is limited by the lack of a non-CRRT-dependent control group. In addition, there is a potential for ascertainment bias in that patients who had vancomycin levels checked may have been more likely to be subtherapeutic than those who did not have levels checked. Even assuming that those without appropriate serum monitoring were therapeutic, we would still have a large proportion of total patients in the subtherapeutic range, however. 

In conclusion, this study demonstrates frequent subtherapeutic blood levels during CVVHD. This is likely due to increased clearance relative to earlier studies or to inadequate dosing. Thus, higher and/or more frequent dosing and more frequent monitoring of vancomycin levels appear necessary. In order to maintain therapeutic levels over larger periods of time and avoid subtherapeutic levels, we recommend an initial dose of 20 mg/kg of vancomycin, followed by 15 mg/kg every 24 h while a patient is on CVVHD at the Qb and Qd utilized, with monitoring of serum levels every 48 – 72 h; “dosing by level” in this setting is not recommended as routine practice. As several studies have failed to demonstrate clinical superiority of CRRT over intermittent hemodialysis in patients with acute kidney injury [1, 7, 8], we postulate that enhanced clearance of antibiotics and perhaps other medications with resulting subtherapeutic levels may actually adversely influence patient outcomes. While pharmacists provide an invaluable resource in the ICU, intensivists often look to the nephrology team to provide drug dosing guidelines during dialytic therapy. An appreciation of drug levels CVVHD will help to make those guidelines more appropriate in this population. 

**Table 1.** Data expressed as mean ± standard deviation except as noted.

Medical intensive care unit (%)	19 (27)
Surgical intensive care unit (%)	51 (73)
Age (y)	57 ± 14
Creatinine at initiation of CVVHD	4.53 ± 2.45
BUN at initiation of CVVHD	69.6 ± 40.4
Albumin	2.28 ± 0.70
Weight (kg)	87.3 ± 25.1
Received vancomycin during hospitalization (%)	66 (94)
Received ≥ 2 doses vancomycin on CRRT (%)	40 (57)

**Figure 1:**
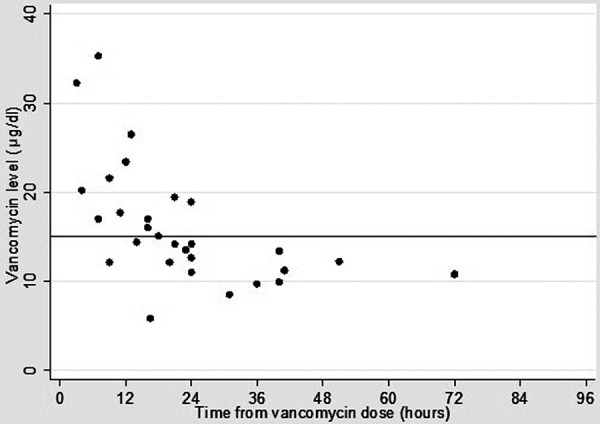
Vancomycin levels (in µg/dl) relative to time since prior administered dose (h). Line indicates therapeutic level threshold.

## References

[1] Rabindranath K Adams J Macleod AM Muirhead N 2007; 3: CD003773. 10.1002/14651858.CD003773.pub3 PMC13199906 17636735

[2] Boereboom FTJ Ververs FFT Blankestijn PJ Savelkoul TJF van Dijk A Vancomycin clearance during continuous venovenous haemofiltration in critically ill patients. Intensive Care Med. 1999; 25: 1100–1104. 10551965 10.1007/s001340051018

[3] DelDot ME Lipman J Tett SE Vancomycin pharmacokinetics in critically ill patients receiving continuous venovenous haemodiafiltration. Br J Clin Pharmacol. 2004; 58: 259–268. 15327585 10.1111/j.1365-2125.2004.02143.x PMC1884563

[4] Sakoulas G Moise-Broder PA Schentag J Forrest A Moellering RC Eliopoulos GM Relationship of MIC and bactericidal activity to efficacy of vancomycin for treatment of methicillin-resistant Staphylococcus aureus bacteremia. J Clin Microbiol. 2004; 42: 2398–2402. 15184410 10.1128/JCM.42.6.2398-2402.2004 PMC427878

[5] Tenover FC Moellering RC The rationale for revising the Clinical and Laboratory Standards Institute vancomycin minimal inhibitory concentration interpretive criteria for Staphylococcus aureus. Clin Infect Dis. 2007; 44: 1208–1215. 17407040 10.1086/513203

[6] Rybak M Lomaestro B Rotschafer JC Moellering R. Craig W Billeter M Dalovisio JR Levine DP. 2009; 66: 82–98. 10.2146/ajhp080434 19106348

[7] Augustine JJ Sandy D Seifert TH Paganini EP A randomized controlled trial comparing intermittent with continuous dialysis in patients with ARF. Am J Kidney Dis. 2004; 44: 1000–1007. 15558520 10.1053/j.ajkd.2004.08.022

[8] Palevsky PM Zhang JH O’Connor TZ Chertow GM Crowley ST Choudhury D Finkel K Kellum JA Paganini E Schein RM Smith MW Swanson KM Thompson BT Vijayan A Watnick S Star RA Peduzzi P Intensity of renal support in critically ill patients with acute kidney injury. N Engl J Med. 2008; 359: 7–20. 18492867 10.1056/NEJMoa0802639 PMC2574780

